# Macromolecular Design and Engineering of New Amphiphilic *N*-Vinylpyrrolidone Terpolymers for Biomedical Applications

**DOI:** 10.3390/ijms242015170

**Published:** 2023-10-14

**Authors:** Svetlana V. Kurmaz, Evgenia O. Perepelitsina, Sergey G. Vasiliev, Irina A. Avilova, Igor I. Khodos, Vladimir A. Kurmaz, Dmitry A. Chernyaev, Yuliya V. Soldatova, Natalia V. Filatova, Irina I. Faingold

**Affiliations:** 1 Federal Research Center of Problems of Chemical Physics and Medicinal Chemistry, Russian Academy of Sciences, 142432 Chernogolovka, Russia; jane@icp.ac.ru (E.O.P.); viesssw@mail.ru (S.G.V.); irkaavka@gmail.com (I.A.A.); kurmaz@icp.ac.ru (V.A.K.); chernyayevda@icp.ac.ru (D.A.C.); soldatovayv@gmail.com (Y.V.S.); natasha55555@yandex.ru (N.V.F.); ifaingold@mail.ru (I.I.F.); 2 Institute of Microelectronics Technology and High-Purity Materials, Russian Academy of Sciences, 142432 Chernogolovka, Russia; khodos.igor@mail.ru

**Keywords:** *N*-vinylpyrrolidone, (di)methacrylates, branching agent, radical polymerization, amphiphilic terpolymer, nanogel, cytotoxicity, D-α-tocopherol, encapsulation, antiradical activity

## Abstract

New amphiphilic VP-(di)methacrylate terpolymers of different monomer compositions and topologies have been synthesized by radical polymerization in toluene without any growth regulator of polymer chains. Their structures and properties in solid state and water solution were studied by double-detector size-exclusion chromatography; IR-, ^1^H, and ^13^C NMR-spectroscopy; DLS, TEM, TG, and DSC methods. The composition of the VP-AlkMA-TEGDM monomer mixture has been established to regulate the topology of the resulting macromolecules. The studied terpolymers presented on TEM images as individual low-contrast particles and their conglomerates of various sizes with highly ordered regions; in general, they are amorphous structures. None of the terpolymers demonstrated cytotoxic effects for noncancerous Vero and tumor HeLa cells. Hydrophobic D-α-tocopherol (TP) was encapsulated in terpolymer nanoparticles (NPs), and its antioxidant activity was evaluated by ABTS (radical monocation 2,2′-azino-bis(3-ethylbenzthiazoline-6-sulfonic acid)) or DPPH (2,2′-diphenyl-1-picrylhydrazyl) methods. The reaction efficiency depends on the TP-NP type. The IC_50_ values for the decolorization reaction of ABTS^•+^ and DPPH inhibition in the presence of initial and encapsulated TP were obtained.

## 1. Introduction

Currently, amphiphilic polymers with complex molecular architecture, in particular, highly branched polymers (HBP) capable of self-assembly, are widely used in developing drug delivery systems [1,2]. They increase their water solubility and bioavailability, provide a long circulation time in the bloodstream, preferential accumulation in inflammatory foci due to increased vascular permeability (EPR effect), and reduce side effects [3,4,5,6]. Possible mechanisms for forming HBP-drug systems have been summarized in [5]. The simplest drug delivery systems are polymer–drug complexes formed due to the capture of small molecules induced by physical interactions with the polymer. However, the number of suitable carriers for these purposes is very limited. Unimolecular micelles based on amphiphilic HBPs stable under temperature/pressure changes are promising candidates owing to their wide possibilities of architecture design and property control. Due to the small size of the internal cavities, they can be loaded with a small number of guest drug molecules. The self-assembling multimolecular micelles with many hydrophobic nuclei can be loaded with a large amount of a drug, which potentially makes them superior delivery systems [5]. Multimolecular micelles are desirable tumor-targeting vehicles due to the EPR effect. Active targeting can be realized by functionalizing micelles with small molecules such as folate, lactose, galactose, and antibodies, which can recognize and bind to specific receptors of cancer cells. So, the drug-loaded multimolecular micelles exhibit enhanced cell inhibition against folic acid receptor-expressing tumor cells [7,8]. It is possible to release smart drug delivery and controlled release of drugs loaded in the responsive micelles by intracellular (pH fluctuation) and external (temperature variation) stimuli. Such environment-responsive HBP micelles can change their volume, structure, or property in response to changes in pH, temperature, ionic strength, etc. [9]. A non-covalent mechanism of drug–polymer interaction is realized in all these systems. In addition, delivery systems have been developed by linking drugs to HBPs through covalent bonds and are known as HBP-drug conjugates [10,11,12,13].

Amphiphilic HBPs have been shown to outperform their linear analogs in delivery systems [14,15,16,17,18]. Their high loading capacity [15], increased stability when coated with gold nanoparticles (AuNPs) [16], and high hemocompatibility [18] were reported. In addition to hydrophobic guest molecules encapsulated in the HBP core, hydrophilic compounds can be fixed on the surface of PNP (polar shell) containing a large number of functional groups, for example, charged biomolecules, or molecules responsible for targeted delivery [19,20].

HBPs can be synthesized by polycondensation [21,22], living radical polymerization [23,24], “click” reactions [25], and copolymerization of commercial mono- and divinyl monomers in the presence of a catalytic chain transfer or a chain transfer agent (CTA) [26,27,28,29,30,31]. The latter has great potential for the synthesis of amphiphilic HBPs as a simple, versatile, and scalable route; it can be readily combined with controlled (CCT) and living radical techniques to achieve improved control over the polymer architecture. The approach applies to a wide range of hydrophilic vinyl monomers. For example, amphiphilic HBPs based on methacrylic acid and ethylene glycol were prepared by Graham et al. [29]; Cambon and co-workers [30] have synthesized amphiphilic HBPs using *N*-isopropylacrylamide as a thermoresponsive monomer. Besenius et al. have synthesized and characterized water-soluble densely branched glyco-polymers [31].

We used the radical polymerization in solution in the presence of 1-decanthiole as traditional CTA [32] or without it [33,34,35,36,37,38,39] to produce amphiphilic VP-dimethacrylate copolymers of various compositions, molecular weights, and architectures at high yields within 2–3 h. Their molecular weight distribution (MWD) and compositional heterogeneity may be improved by fractionation. A controlled radical polymerization technique, such as RAFT, is more effective for the synthesis of a compositionally homogeneous copolymer with a narrow MWD. However, problems arise in choosing a suitable RAFT agent, reaction conditions, and the incorporation of the RAFT agent into polymer chains and its effect on the toxicity of produced polymers. Before [40], branched *N*-vinyl-2-pyrrolidone copolymers were synthesized in several stages: first, linear VP terpolymers with crotonic acid and 2-hydroxyethyl methacrylate were obtained, then they were cross-linked with 1,6-hexamethylene diisocyanate at the primary hydroxyl groups, and the resulting network structures were subjected to hydrolysis in order to be used as carriers of commercial antibiotics.

Our studies have shown that amphiphilic copolymers of *N*-vinylpyrrolidone with complex macromolecular architecture can be of interest to medicine, pharmaceutics, and cosmetic applications. They are characterized by pronounced amphiphilicity that can be controlled easily via comonomer nature and copolymer composition and the ability of copolymers of some composition to respond to external conditions (temperature and pH medium) and hydrolysis in acidic and alkaline media along C–O bonds of dimethacrylate units; they have a sufficiently small size of individual macromolecules and their aggregates in water are able to facilitate their efficient cellular and tissue uptake. Some of these features are not inherent to linear PVP, and they determine the physicochemical advantages of branched VP copolymers.

Based on amphiphilic VP-copolymers, we have created nanosized systems of lipophilic organic complexes of platinum(IV) with antitumor activity [33,34], water-insoluble photosensitizers like zinc tetraphenylporphyrinate to visualize their intracellular accumulations [35,36], and methyl pheophorbide a for the photodynamic therapy [37]. The high biocompatibility of polymer carriers and their ability to penetrate into Vero and HeLa cells have been demonstrated in vitro, which indicates that the developed copolymers are promising for the intracellular delivery of commercial drugs and original compounds with biological activity. In in vivo experiments, the VP-copolymer exhibits no toxicity [38].

Modification of such copolymers with even a small amount of hydrophilic methacrylic acid (MAA) or poly(ethylene glycol) methyl ether methacrylate (PEGMEM) monomers, as shown by our studies [35,36], can significantly change their molecular weight, polydispersity, amphiphilicity, self-assembly, and response to external stimuli (temperature and pH medium). Hydrophobic comonomers, e.g., higher alkyl methacrylates (AlkMA), in turn, can also be useful for the amphiphilicity of VP-TEGDM copolymers varying and the increasing efficiency of encapsulation of poorly water-soluble or -insoluble biologically active compounds.

**Figure 1:**
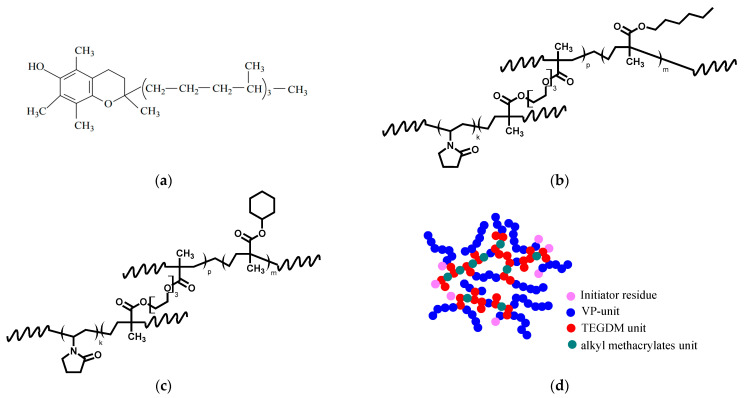
Chemical structure (**a**) of TP, (**b**) VP-HMA-TEGDM, and (**c**) VP-CHMA-TEGDM terpolymers, and (**d**) one of their possible topological structures of branched macromolecules.

In work [39], we have prepared water-soluble forms of D-α-tocopherol (TP) (Figure 1a) as an effective antioxidant by encapsulating into the nanoparticles (NPs) of an amphiphilic copolymer of *N*-vinylpyrrolidone with triethylene glycol dimethacrylate and amphiphilic terpolymer of *N*-vinylpyrrolidone with hexyl methacrylate and triethylene glycol dimethacrylate, and their high antioxidant activity has been established by the thiobarbituric acid reactive species (TBARS) and chemiluminescence assays. They effectively inhibited the process of spontaneous lipid peroxidation as well as initial TP and exhibited antiglycation activity [41] against vesperlysine and pentosidine-like AGEs. We concluded that the developed TP-NPs are promising materials with antioxidant and antiglycation activity, and further investigation of these nanosized systems is needed to optimize their performance.

Therefore, the goal of this work is to synthesize the series of amphiphilic VP terpolymers with higher alkyl methacrylates as typical hydrophobic comonomers—hexyl methacrylate (HMA) or cyclohexyl methacrylate (CHMA) and triethylene glycol dimethacrylate (TEGDM) as branching agents of different compositions—and to study their structure/properties in solid state/water solutions, cytotoxicity on normal Vero and tumor HeLa cells, and the antiradical activity of TP encapsulated in terpolymer by ABTS and DPPH methods where ABTS and DPPH are the monocation radical of 2,2′-azino-bis(3-ethylbenzthiazoline-6-sulfonic acid) and 2,2′-diphenyl-1-picrylhydrazyl, respectively, due to their wide distribution, accuracy, and without tissue or cell homogenates applications to avoid any interactions with the components of homogenates.

## 2. Results and Discussions

### 2.1. Structure and Characteristics of N-Vinylpyrrolidone with (Di)methacrylates Terpolymers

The terpolymers were obtained in toluene in the absence of any polymer chain growth regulator at various molar ratios of VP:AlkMA:TEGDM comonomers (Table 1).

Terpolymers with chemical structures shown schematically in Figure 1b,c were synthesized in toluene by radical polymerization of VP with (di)methacrylates containing one (HMA and CHMA) or two reactive C=C bonds (TEGDM). One of the double bonds of the bifunctional comonomer was involved in the growth of the main chain, while the other formed side branches that appeared when polymeric radicals were attached to the “pendant” C=C bond of TEGDM. At the initial stage, all radicals add more reactive methacrylic monomers, statistically distributed in growing chains, and copolymers enriched by these units are formed [32]. Thus, random terpolymers are formed at the early stages of copolymerization. At deep stages of copolymerization, as follows from the kinetic curves of copolymerization of VP with dimethacrylate [32], PVP radicals attach to the “pendant” C=C bonds of dimethacrylate units. As a result, a 3D structure is formed with moieties consisting of branching AlkMA and TEGDM fragments and linear PVP chains. (Di)methacrylates moieties in a polymer chain can be branched or slightly cross-linked if intramolecular cyclization takes place due to the reaction of the growing polymer radical with the pendant C=C bond of the TEGDM units of the same polymer chain. AlkMA monomers with bulky alkyl substituents create steric hindrances to the intramolecular cross-linking reaction in primary polymer chains, thereby preventing the formation of highly cross-linked nanogel structures. Figure 1d shows the possible topological structure of branched VP-AlkMA-TEGDM macromolecules.

**Figure 2:**
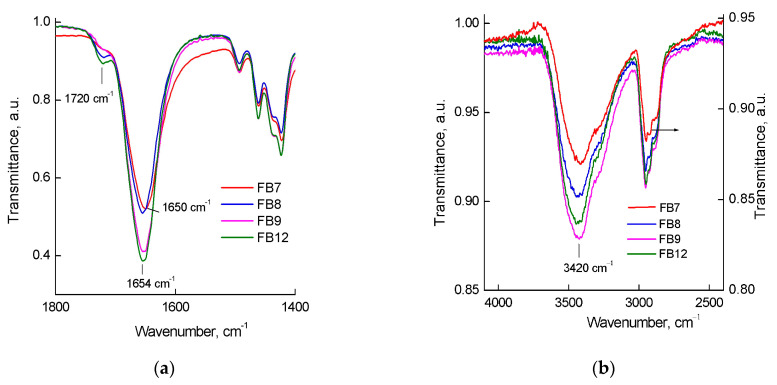
IR spectra of studied terpolymers in the regions (**a**) of 1800–1400 cm^−1^ and (**b**) 4000–2500 cm^−1^.

The studied terpolymers were characterized by IR spectroscopy. In their spectra, characteristic absorption bands at wavenumbers ~1720 cm^−1^ and in the regions of 1654–1650 cm^−1^ corresponding to the stretching vibrations of C=O groups in (di)methacrylates and VP units, respectively, were observed. These bands are well separated from each other, as can be seen in the IR spectra (Figure 2a). The absorption band of (di)methacrylate units depends on their content in terpolymers. FB8 and FB12 absorption bands are more pronounced and intense compared with other terpolymers. The position of the C=O groups of the VP units’ absorption band depends on the type of polymer. For FB7, it is shifted to lower wavenumbers, probably as a result of stronger binding to adsorbed water molecules. In our work [42], we have shown that the absorption band position of stretching vibrations of the C=O bond of the lactam cycle of VP units depended on the amount of adsorbed water and the degree of its bonding with a polymer. The broad absorption band typical for the stretching vibrations of OH groups of adsorbed water bonded by hydrogen bonds with the amphiphilic terpolymers is at 3600–3000 cm^−1^ (Figure 2b).

**Figure 3:**
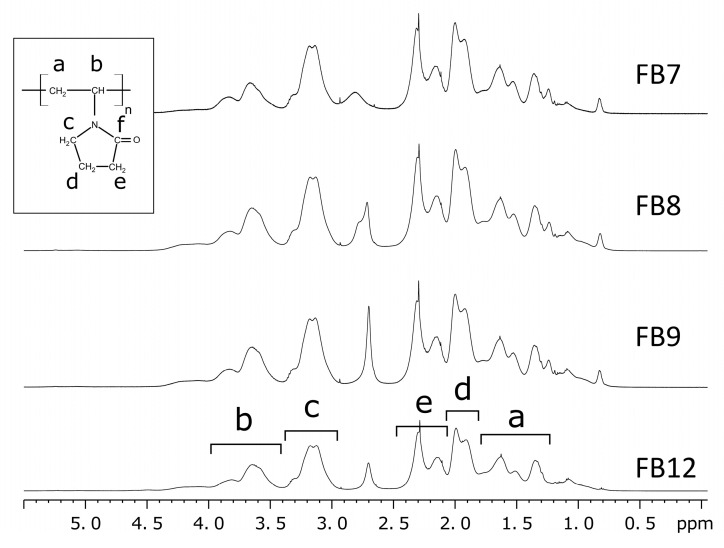
^1^H NMR spectra of terpolymers obtained in CDCl_3_ solutions.

The ^1^H NMR spectra of terpolymers are shown in Figure 3. The main signals were ascribed to the PVP units constituting a major portion of terpolymers. The main chain methylene (a) and methine (b) signals appear at δ1.27–1.8 and δ3.4–4.4 ppm, respectively. The signals of the protons belonging to the side-chain methylene carbons are observed at δ3.14–2.95 (c), δ1.8–2.05 (d), and δ2.05–2.5 ppm. Additional methylene protons due to the TEGDMA, HMA, and CHMA copolymer units overlap mainly with signals (a) and (b), giving rise to the shoulders on their left and right sides, respectively. The signal of the aliphatic methyl group of HMA appears separately at ca. δ0.83 ppm. This signal is absent for terpolymer FB12 containing only CHMA units. The signals attributed to the C=CH_2_ were not observed, showing a high conversion for these groups. The signal at δ2.6–2.9 that does not appear in carbon-detected HSQC experiments was attributed to the protons of water molecules.

**Figure 4:**
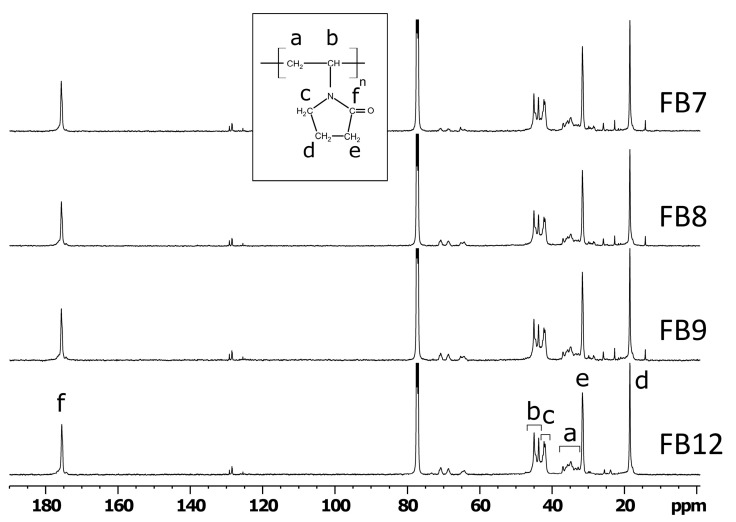
^13^C NMR spectra of terpolymers in CDCl_3_.

The main signals belonging to the VP units in ^13^C NMR spectra of all four terpolymers are quite similar, as seen in Figure 4. The signals of the main chain CH_2_ (a) and CH (b) units broadened due to the various configurational sequences [43] appear at δ32.3–38 and δ43.2–46 ppm, respectively. The ring side-chain carbons show narrower signals of methylene groups at δ18.41 (d), δ31.57 (e), δ42.2 (c) ppm, and carbonyl group at δ175.64 ppm. Signals near δ130 ppm belong to the traces of toluene presented in the samples. The signals of toluene are also present in the aromatic region of ^1^H spectra (not shown) and aliphatic region (small narrow signal of methyl group overlapping with (e) signal of VP in Figure 4).

The small signal at δ73.3 ppm specific only to the sample FB12 was assigned to the OCH group of CHMA. For this compound, the signal at δ14.7 is absent. This signal corresponds to the aliphatic methyl group of HMA for other polymers. The signals at δ22.65 and δ25.78 were attributed to the methylene groups in α and β positions to the methyl group of the aliphatic tail in terpolymers FB7, FB8, and FB9. For FB12 with cyclohexyl units, these signals are located closer to each other at δ23.8 and δ25.47, respectively. The signals at δ70.8 and δ68.7 ppm were attributed to the methylene groups in α and β to the methacrylate group in TEGDM, as shown in Figure S1. The signal of the central methylene groups of the TEGDM (c in Figure 3) overlaps with the signal of the O–CH_2_ group of HMA (d in Figure S1). The signal (d) is absent for the sample FB12 since it contains an O–CH group instead, which is labeled as (e) in Figure S1. The rest of the methylene groups are observed on the right of the signal (e) (Figure 4) in the range δ27.8–30.3 ppm. As noticed for ^1^H spectra earlier, no signals that can be attributed to the C=CH_2_ groups were observed.

For estimating the composition of terpolymers, the region of O–CH_2_ and O–CH groups is of particular interest. The signal enhancement due to the nuclear Overhauser effect is expected to be similar for all terpolymers. Thus, the relative intensities of the lines can be used to determine the composition. The signals used for the calculations are labeled in Figure S1. We used a dedicated deconvolution program for the accurate determination of the intensities of the lines [44]. From the ratio of the lines *x* = (a + b)/(c + d), one can find the desired ratio of HMA/TEGDM units in terpolymers as (4*x* − 2). For the terpolymer FB12 with CHMA units, no overlapping of the signals c and d occurs (signal d is absent, and all lines are exclusively due to TEGDM), so the ratio *x* should closely correspond to ½. The calculated value *x* = 0.50 for FB12 confirms the appropriateness of the chosen method for composition determination. For terpolymer FB12, the ratio of CHMA/TEGDM units was calculated from the ratio of the separate OCH signal of CHMA to the sum of the signals a and b (Figure S1) originating from TEGDM units. Considering the measurement errors (5−10%), the experimental values of HMA/TEGDM and CHMA/TEGDM were 1.05 and 0.37 in FB7 and FB12 terpolymers, respectively, and coincide with the ones in the reaction mixtures (Table 1). Thus, the content of (di)methacrylates having the same reactivity in these terpolymers corresponds to their concentration in comonomer mixtures. Meanwhile, the HMA/TEGDM ratio in FB8 and FB9 terpolymers is 0.25 and 0.38, i.e., lower than the ones in the corresponding reaction mixtures; however, the trend of their change is quite correct.

Table 1 shows the nitrogen (%) content of VP units and the comonomer compositions (mol%) of the terpolymers calculated from them. Here, only the total content of AlkMA and TEGDMA units is indicated. It can be seen that the inclusion of AlkMA units in macromolecules leads to a decrease in their nitrogen content and an increase in their monomer units in the polymer chains. It follows from the above data that the content of (di)methacrylates in the studied terpolymers varies from ~3 to ~10 mol%, depending on the composition of the monomer mixture.

Molecular weight distribution curves are shown in Figure S2. For FB9 and FB7 terpolymers, they are close to the PVP curve. There is a second peak in the region of high molecular weights of FB8 and FB12 curves, probably corresponding to the fraction of highly branched macromolecules. Table 2 contains absolute values of the molecular masses *M*_w_ and *PD* of the initial terpolymers, purified by reprecipitation, derived from the double-detection SEC data. It follows from the data presented that the FB9 and FB7 copolymers have *M*_w_ and PD comparable to the linear PVP (*M*_w_ ~ 65 kDa, PD = 2). The molecular weight characteristics of FB8 and FB12 differ significantly from them and indicate the branched nature of these macromolecules. It is known that branched polymers synthesized by this method are characterized by high molecular weight and polydispersity since they represent a mixture of macromolecules with different molecular weights and architecture [27]. For example, a copolymer of MAA with divinylbenzene synthesized under conditions of chain transfer to dodecanethiol at a molar ratio of 100:2:1 is characterized by a *PD* value of ~55 [45]. Chain growth during the formation of FB9 and FB7 is limited, probably as a result of the inefficient consumption of C=C bonds of TEGDM in the intramolecular cross-linking reaction, which leads to the formation of small cycles in the copolymer structure and the formation of nanogel particles. Due to their small size, the molecular weight of terpolymers can also be estimated by SEC. The absolute molecular masses *M*_w_ and *PD* values of the purified terpolymers change insufficiently (Table 2).

**Figure 5:**
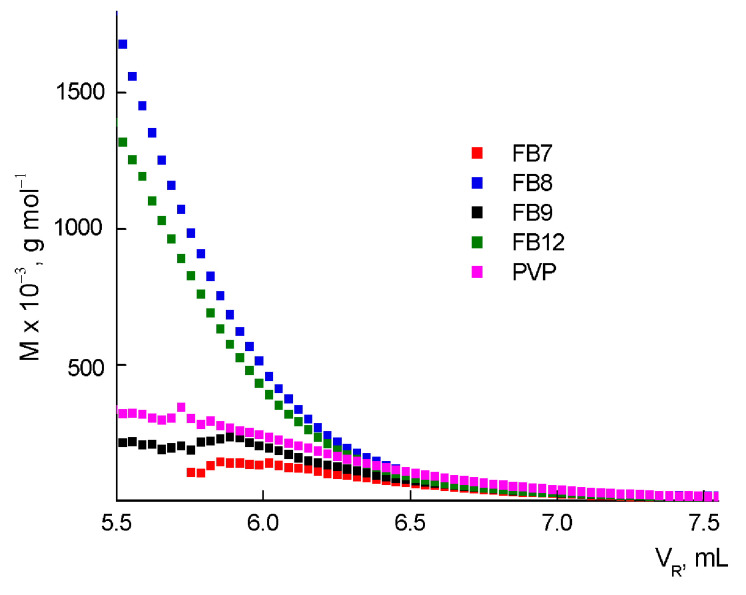
Dependencies of the molecular weight *M* on the eluent volume *V*_R_ for the terpolymers and PVP.

To analyze the topology of macromolecules, we used the dependencies of the molecular weight *M* on the eluent volume *V*_R_ (Figure 5) for the synthesized terpolymers and linear PVP and their MALS traces (Figure S3a). It can be seen that these dependencies for FB9 and FB7 in the *V*_R_ range of ~5.5–6.25 mL are lower than for linear PVP, while for FB8 and FB12, they are much higher. This means that copolymers eluted at given *V*_R_ values have different packing densities of polymer chains compared to linear PVP and among themselves. It can be assumed that FB9 and FB7 are cyclic structures that swell poorly in MP as eluent. In comparison with FB9, FB7 has a higher degree of intramolecular cross-linking. Perhaps the result should be interpreted as the formation of nanogel particles, which contain cycles due to intramolecular cross-linkings. FB8 and FB12 copolymers with the same eluent volume *V*_R_ have a significantly higher molecular weight in the range *V*_R_ = 5.5–6.25 mL. This means that macromolecules of a given size and molecular weight have a denser molecular packing of polymer chains, i.e., they are branched compared to FB9 and FB7. This is supported by the dependencies rms(*M*) and rms(*V*_R_) (Figure S3) for linear PVP and FB9 and FB8 terpolymers. It can be seen that their rms values are, respectively, higher and lower than those of PVP in this range of molecular weights. From the obtained dependencies, the Zimm factor can be estimated as the ratio rms of the given copolymer and linear PVP for FB8 macromolecules with *M*~10^5^, which is ca. 0.7. Thus, an increase in TEGDM content in the monomer mixture leads to an increase in the molecular weight and polydispersity of terpolymers and the formation of branched macromolecules.

### 2.2. Behavior of VP-AlkMA-TEGDM Terpolymers in Water

The studied terpolymers consist of units of a hydrophilic monomer, VP, and hydrophobic HMA, CHMA, and TEGDM comonomer units. To achieve thermodynamic stability by reducing the hydrophobic interaction of non-polar or low-polar groups and water, amphiphilic macromolecules take the form of a core–shell, where the hydrophobic moieties form a core, around which there is a hydrophilic outer shell from PVP chains [42]. At certain concentrations in water, as a result of self-assembly, they can form aggregates as multimolecular micelles like other amphiphilic HBPs [5].

In this regard, the critical concentrations of aggregation of studied terpolymers were determined in water by the dependency of the average light scattering intensity *I* on the concentration of terpolymers *C* (Figure S4). As a result of their approximation by two linear dependencies, CAC values were obtained (Table 2), which depend on the terpolymer’s composition, molecular weight, and topology. Terpolymer FB7 is the most prone to aggregation among the studied copolymers and has a lower CAC value. As a consequence, its dilute aqueous solutions became strongly opalescent at room temperature. Below the CAC, macromolecules with a 3D structure are presented mainly in the form of monomolecular micelles; near the CAC, their aggregates appear as multimolecular micelles stabilized by hydrogen bonding with water; their content in the solution increases with an increase in the terpolymer concentration.

**Figure 6:**
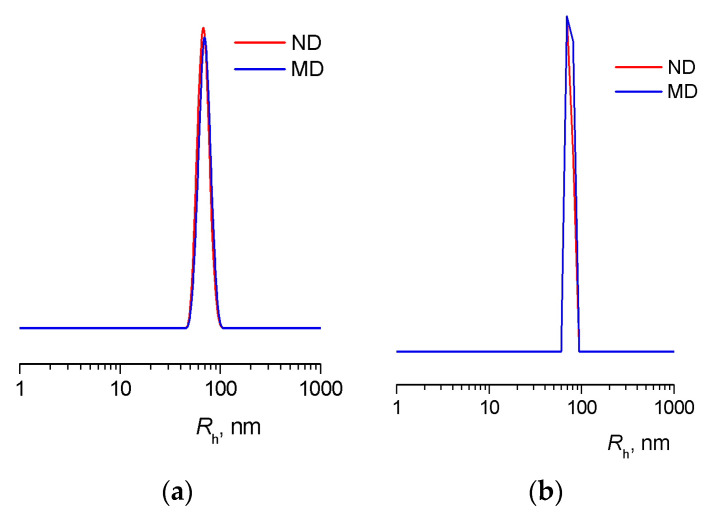
Mass (MD) and number (ND) distribution on particle size (**a**) in FB9 and (**b**) FB7 water solution. [FB9] = 0.62 mg mL^−1^, [FB7] = 0.31 mg mL^−1^. *T* = 30 °C.

Mass and number distributions of particle size in FB9 and FB7 water solutions are unimodal (Figure 6), and the coefficients of polydispersity of the particles in analyzed solutions are equal to 1. The particle size distribution practically does not change with increasing temperature (Figure S5).

**Figure 7:**
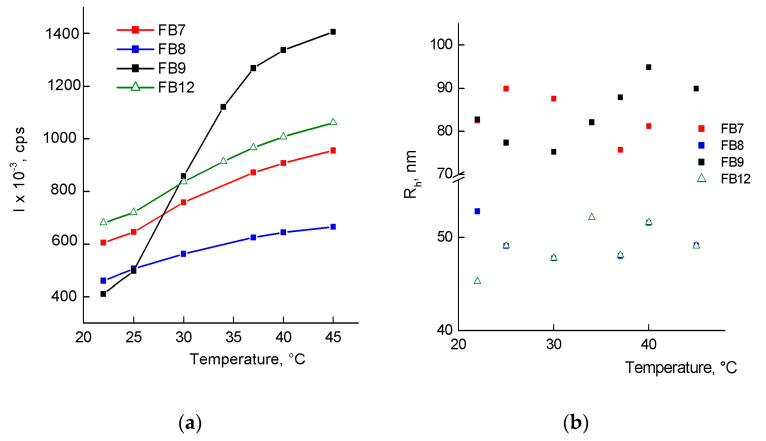
Dependencies (**a**) of the average light scattering intensity *I* and (**b**) the average hydrodynamic radius *R*_h_ of scattering centers of FB9, FB7, FB8, and FB12 water solutions on temperature.

Figure 7a shows dependencies of the average light scattering intensity *I* on temperature in the range of 22–45 °C. The shape of *I*(*T*) curves strongly depends on terpolymer composition and its molecular weight. A sharp *I*(*T*) curve is specific for the FB9 solution, in contrast to the others. Terpolymer FB9 has an LCST value in the range of 30–40 °C in the water solution, and this solution became cloudy reversibly. Previously, we have shown that VP terpolymers with MAA units were thermally sensitive, and *I*(*T*) dependencies were S-shaped [35]. Below LCST, no phase separation occurs at any polymer concentration in the solution. As a rule, it is observed in systems with strongly interacting components between the molecules in which hydrogen bonds can form [28,45,46]. These dependencies are weakly pronounced for copolymers FB7, FB8, and FB12. In water solutions of FB7, FB8, and FB12, the LCST value was probably shifted strongly to the low-temperature region, and as a result, only their high-temperature regions of the S-shaped curve were visible in Figure 7a. Polymers with LCST in the physiological temperature range are of interest as “smart” BAC delivery vehicles [5]. The collapse of the polar shell and the growth of hydrophobic interactions induced by temperature will lead to the release of BAC from the NPs at the LCST temperature and above.

The *R*_h_ values of aggregates of FB9 and FB7 copolymers (nanogels) are almost two times higher than those of branched FB8 and FB12 copolymers (Figure 7b), which is due to the different content of TEGDM units in their macromolecules and their topological structures. Moreover, the sizes of scattering centers in water solutions of FB8 and FB12, measured in the range of 22–45 °C, coincide, i.e., the structure of the AlkMA substituent (aliphatic or cyclic alkyl substituent) does not affect their dynamic parameters. The determining factor seems to be the content of TEGDM in the polymer chains and the related topology of the macromolecule.

### 2.3. TEM Analysis of the Terpolymers’ Structure

**Figure 8:**
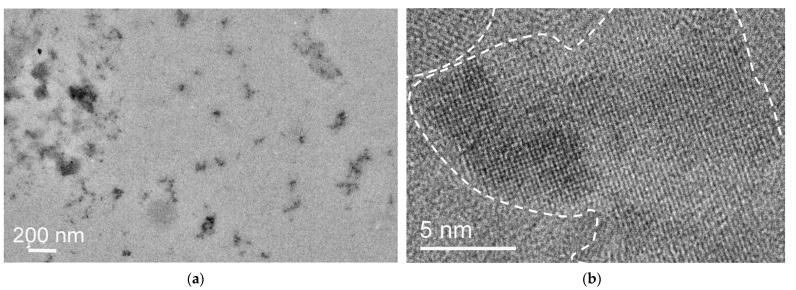
TEM images of FB7 terpolymer: (**a**) low magnification image; (**b**) direct lattice resolution of highly ordered regions in nanoparticles. The boundaries of the highly ordered region are shown by dotted lines.

**Figure 9:**
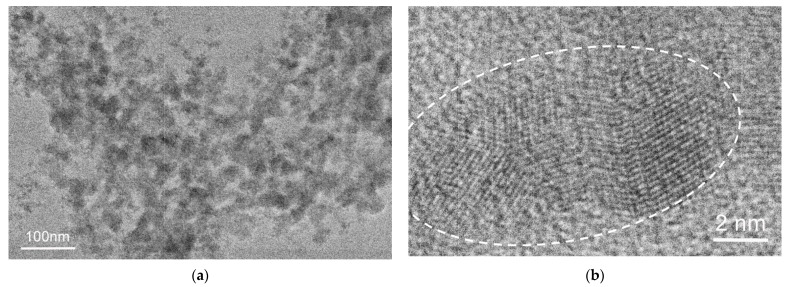
The TEM images (**a**) of FB8 nanoparticles obtained from a filtered water solution and (**b**) of the highly ordered region of coagulated nanoparticles (marked with dotted oval) from an initial solution.

**Figure 10:**
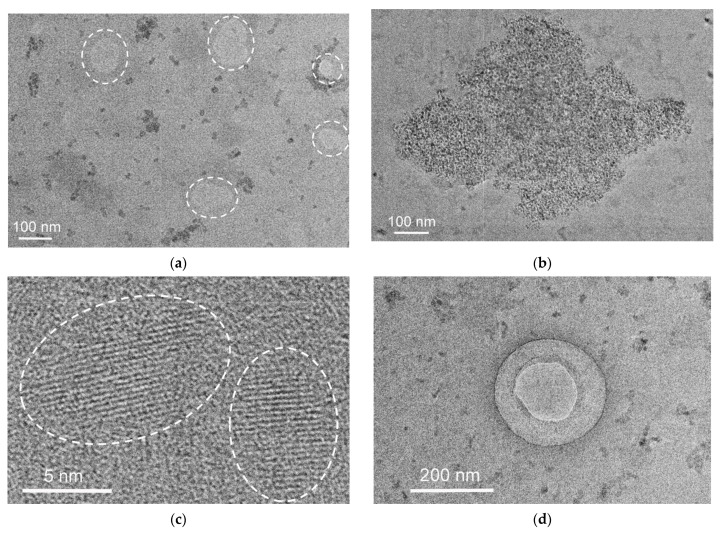
TEM images (**a**,**b**) of FB12 nanoparticles of different sizes and forms, (**c**) highly ordered regions in a nanoparticle, and (**d**) circular formation (all mentioned features of the sample structure are marked with dotted lines).

Figure 8, Figure 9 and Figure 10 show images of FB7, FB8, and FB12 terpolymers obtained from water solutions. Both individual low-contrast particles and their variously sized conglomerates of FB7 terpolymer are visible in Figure 8a. The boundaries of individual nanoparticles and their clusters are indistinct and often indistinguishable in conglomerates. In addition, some number of nanoparticles with highly ordered regions were found (Figure 8b). The performed analysis of the FB7 images and electron diffraction patterns allows us to conclude that the sample is an amorphous polymer with highly ordered regions.

TEM images show that “elementary” nanoparticles of FB8 are larger than those of FB7 (20–40 nm), and they form flatter clusters (Figure 9a). No highly ordered regions were found in the studied filtered sample, but they were present in the initial sample (Figure 9b).

The individual FB12 particles are ca. 10 nm in size, and most of them form polymer clusters up to 100 nm in size (Figure 10a); however, they may also form clusters up to 1 micron in size (Figure 10b). Numerous areas with weak contrast of significant size were observed (Figure 10a). TEM images show the presence of highly ordered regions, similar to the FB7 and FB8 terpolymers, despite the difference in their physicochemical characteristics and macromolecule structure. Figure 10c shows nanoparticles with highly ordered regions of about 6 and 8 nm in size. The existence of such regions is also proved by diffraction patterns. In the FB12 terpolymer, there are circular formations about 0.2 microns in size (Figure 10d), which are similar to those observed in the unfiltered FB7 sample [39].

Thus, all the studied polymers are mainly amorphous materials; nevertheless, highly ordered regions are detected in them. This may be due to the conformation of hydrated PVP chains, which make up the majority of these terpolymers. We showed [42] that water forms complexes with VP units in such copolymers with different stoichiometry and energy similar to linear PVP. It was found in [47] that covalently bonded to the main aliphatic chain pyrrolidone rings or rather only the axes of those rings are located in parallel planes in “dry” PVP. As a result of electrostatic interaction, pyrrolidone rings diverge “fan” to the maximum possible distance *r*_av_ = 4.622 Å, which determines the helical conformation of polymer chain sections in this PVP. Meanwhile, in a hydrated polymer, the first water molecule already forms two H-bonds with the oxygen atoms of two pyrrolidone rings. As a result, the distance between the rings decreases to ~4.2 Å. The pyrrolidone rings’ convergence leads to the straightening of the chain sections upon interaction with water, which is confirmed by the X-ray diffraction studies of hydrated and water-dried PVP [47].

### 2.4. Thermophysical and Thermochemical Properties of VP-AlkMA-TEGDM Terpolymers

It is known [48,49] that the *T*_g_ value of dendrimers and HBPs depends not only on *M*_w_ but also on the number of end groups in the macromolecule. Commonly, it is lower than linear and depends on the chemical nature of the end groups [50,51]. It is suggested that, in contrast to linear polymers, the glass transition of dendrimers and hyperbranched polymers is associated not with the limitation of segmental mobility but with the freezing of motion of macromolecules [52].

The *T*_g_ value of linear PVP with a molecular weight of ~10^3^ kDa depends on the degree of hydration, and it lies in the range of 168–179 °C [47]. PVP with *M*_w_~65 kDa was characterized by a *T*_g_ value ca. 115 °C. In VP-dimethacrylate copolymers obtained under chain transfer conditions [53], we have found two glass transition temperatures related to defrosting of the segmental mobility of branched polymethacrylate chains and linear PVP chains in their macromolecules. Here, it was possible, with great difficulty, to find glass transition areas in some of the studied terpolymers because weakly pronounced steps of the change in heat capacity with temperature were observed near 110–120 °C on DSC curves (Figure S6). This did not allow us to unambiguously determine the *T*_g_ values of the studied terpolymers, but we can conclude that the movement of such macromolecules is partially or completely frozen and does not depend on their physical and chemical parameters and structure.

**Figure 11:**
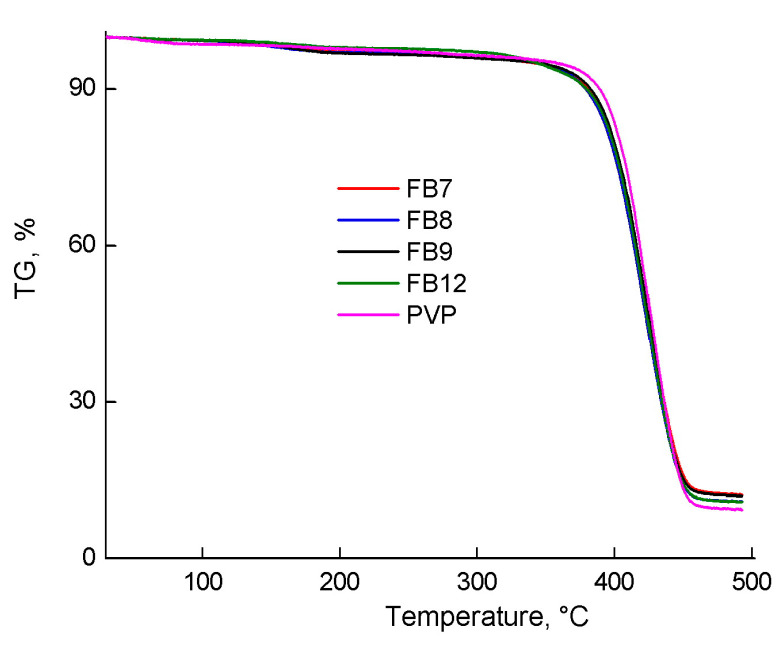
TG curves for the studied terpolymers and PVP in the temperature range of 40–500 °C.

Figure 11 shows TG curves for the studied terpolymers in the temperature range of 40–500 °C. The main weight loss of copolymers occurs at temperatures above 300 °C and ends at about 450 °C; about 10% of the product does not decompose under these conditions. The weight loss is caused by the thermolysis of polymethacrylate and PVP chains. Increasing the proportion of thermally less stable methacrylate units in their composition does not change the thermal stability. Note that PVP itself shows greater thermal stability than VP-HMA-TEGDM terpolymers at temperatures above 150 °C. Figure S7 shows TG curves of terpolymers in the initial sections with a weight loss of up to no more than 95–96%. PVP loses mass earlier than the studied terpolymers. As a hydrophilic polymer, it contains more water that is removed as the temperature rises. FB12 is the most stable due to the CHMA units contained in its polymer chains. The weight loss of 2–3% of the investigated copolymers in the region of 110–200 °C is caused by the removal of water, which is strongly associated with the polar groups of the terpolymer. Overall, VP-AlkMA-TEGDM terpolymers demonstrate similar thermal stability, practically independent of their composition, molecular weight, and topological structure.

### 2.5. Cytotoxicity of VP-AlkMA-TEGDM Terpolymers for Noncancerous and Tumor Cells

**Figure 12:**
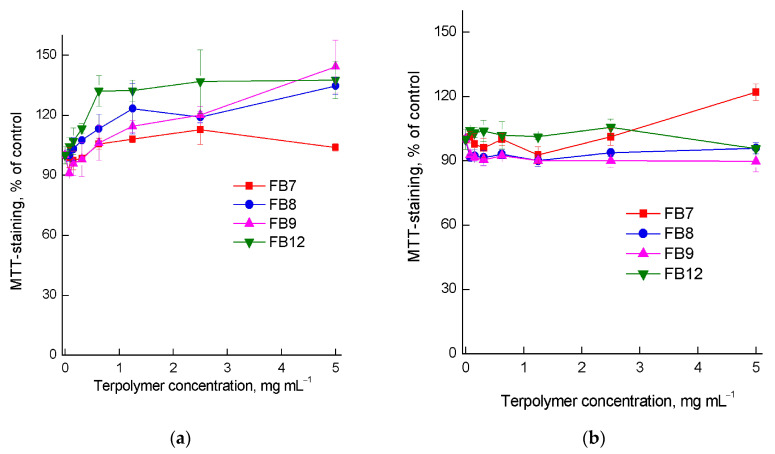
Dose–response curves for (**a**) Vero and (**b**) HeLa cells treated with FB7, FB8, FB9, and FB12 terpolymers for 72 h.

The studied terpolymers might be promising as carriers and delivery vehicles for drugs. As possible carriers, they must have low toxicity to cells and tissues of the body. In this regard, we studied the cytotoxic properties of terpolymers in vitro on mammalian cell models using the MTT assay. Figure 12 shows the dose–response curves for FB7, FB8, FB9, and FB12 terpolymers for noncancerous Vero and tumor HeLa cells within 72 h. It can be seen that FB7, FB8, FB9, and FB12 terpolymers in the entire range of concentrations explored do not reduce the viability of Vero and HeLa cells. Thus, the studied terpolymers are low in toxicity in the entire range of concentrations explored, which allows their use in biomedical applications.

### 2.6. Encapsulated in Terpolymers α-Tocopherol and Evaluation of Its Antiradical Activity by ABTS and DPPH Methods

The studied terpolymers based on biocompatible *N*-vinylpyrrolidone can be of interest as new platforms for fat-soluble α-tocopherol to increase its solubility and bioavailability. TP is the most abundant and effective form of vitamin E [54], which is a chain-breaking antioxidant in human plasma and tissues [55,56]. It effectively scavenges free radicals of unsaturated lipids by transferring the phenolic H-atom to a propagating peroxyl or alkoxyl radicals giving a non-radical lipid product and the α-tocopheroxyl radicals that react with second free radicals or each other to form a non-radical product [57].

Here, TP was encapsulated in NPs based on FB7, FB8, and FB12 and PVP by a simple and effective method of direct dissolution in two steps, as described in Section 3.2. In the first step, solutions of the terpolymers and TP in isopropyl alcohol were used to maintain the uniformity of the medium and prevent self-association of the reagents, and homogeneous and transparent solutions were produced. After evaporation of the common solvent for the reagents at room temperature, transparent films with distributed drugs in a terpolymer matrix were obtained. In the second step, the dry films were solved in water at room temperature. At ~1–2 mg mL^−1^ concentration, these solutions were clear or slightly opalescent in the case of FB7-TP.

**Figure 13:**
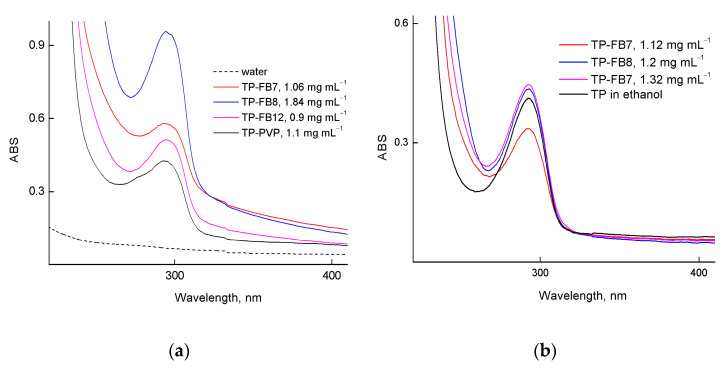
Absorption spectra (**a**) of TP-FB7, TP-FB8, TP-FB12 and TP-PVP in water and (**b**) of TP-FB7, TP-FB8, TP-FB12 and TP in ethanol; cuvette is 1 cm.

Figure 13a shows the absorption spectra of TP-FB7, TP-FB8, TP-FB12, and TP-PVP in water. In spectra, there is a TP absorption band at ~294 nm due to its solubilization by the amphiphilic terpolymers and linear PVP. This suggests that the TP molecules in all NPs have a similar local environment. However, the absorption band has a more complex shape in the TP-PVP spectrum, which may be due to the free and bound form of tocopherol in this polymer. To prove the efficiency of TP encapsulation in terpolymers, we analyzed the absorption spectra of water solutions and compared them with control experiments performed in the absence of a terpolymer, as in [39]. The absorption spectrum of the solution in the control experiment (without terpolymer) has no typical TP band at 280–300 nm. Therefore, we consider that all TP molecules are encapsulated into NPs, and the efficiency of TP encapsulation is ca. 100%.

Unlike water, TP-FB7, TP-FB8, and TP-FB12 dissolve readily in ethanol, and there is a characteristic TP absorption band at ~292 nm in the absorption spectra (Figure 13b). The dependency of its optical density on the TP concentration (10^−4^–10^−5^ M) is linear, and the molar extinction coefficient coincides with the same value for free TP. Using the TP calibration dependency and the concentration dependency of TP-NPs in ethanol, we confirmed the full encapsulation of the compound in NPs.

TP molecules can be physically entrapped with the NPs and localized in the polar shell, forming, according to quantum chemical modeling [39], the hydrogen bond to the oxygen atom of the lactam ring of the VP units. In addition, interactions of the oxygen atoms of both ether and ester groups of TEGDM and HMA units of low polar moieties of polymer chains with TP can occur. Thus, TP binds to terpolymers through intermolecular interactions, primarily hydrogen bonding, which is formed between the OH group of TP and donor groups of terpolymers.

**Figure 14:**
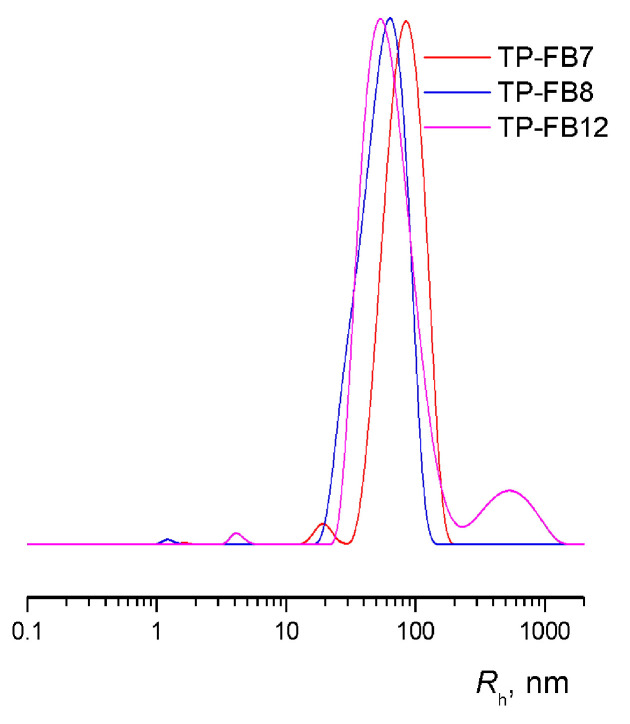
DLS curves of TP-FB7, TP-FB8, and TP-FB12 in water at 25 °C and concentrations of 0.5 mg mL^−1^.

According to DLS data (Figure 14), the *R*_h_ values of TP-FB7, TP-FB8, and TP-FB12 were ~90, 57, and 57 nm, respectively, in concentration regions of 0.5–0.06 mg mL^−1^ in water solution at 25 °C. In TP-FB7, TP-FB8, and TP-FB12 solutions (0.5 mg mL^−1^), the average intensity of light scattering reached high values (~10^6^ cps). Meanwhile, these systems were readily soluble in ethanol, as evidenced by the low average intensity of light scattering (~10^4^ cps). At the same time, small scattering centers were found in solutions of TP-FB7, TP-FB8, and TP-FB12 (1, 1.3, and 1.2 mg mL^−1^, respectively). Thus, particles of *R*_h_ about 5 nm were found in the alcohol solution of TP-FB7, while scattering centers with *R*_h_ about 17 and 20 nm were mainly present in the TP-FB8 and TP-FB12 solutions.

**Figure 15:**
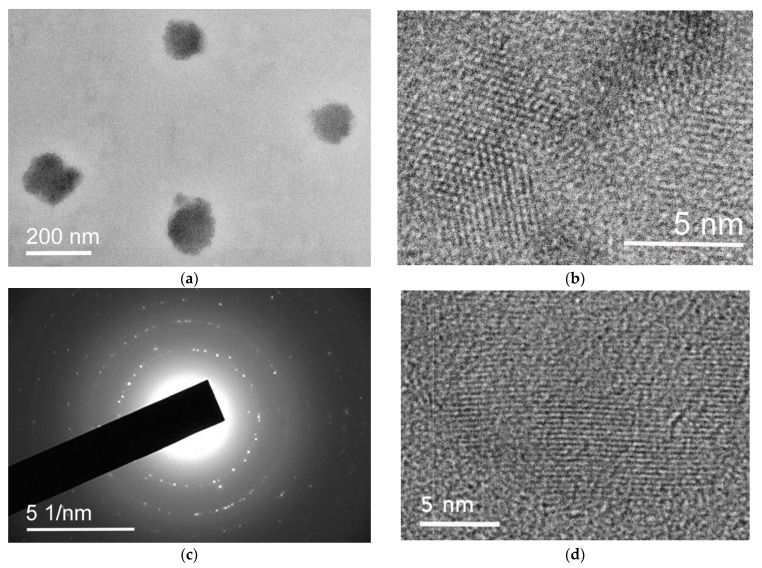
TEM images (**a**) of typical amorphous TP-FB7 nanoparticles; (**b**,**d**) lattice resolution images of regions with a periodical structure; (**c**) selected area diffraction pattern showing the periodicity in the sample structure.

An example of TEM images of TP-FB7 nanoparticles is shown in Figure 15. Most of the particles, including those shown in Figure 15a, are amorphous, but there are also a number of particles containing ordered regions. High magnification images (Figure 15b) clearly demonstrate the presence of periodicities 5–15 nm in size. The presence of ordered regions in the sample is also evidenced by diffraction spots on the selected area diffraction pattern in Figure 15c. Note that the ordered regions can be both three-dimensional and depicted with strong contrast (Figure 15b) and, apparently, single-layered, at least to a significant extent. Figure 15d shows such an ordered region with very weak periodic contrast.

In the IR spectrum of TP (Figure S8a), there is a broad absorption band of stretching vibrations of the OH-group in the region of 3700–3100 cm^−1^ that indicates its participation in intermolecular hydrogen bond formation. Some of the OH groups of TP remain free, which is proved by the narrow absorption band at 3630 cm^−1^. The formation of intermolecular hydrogen in the TP hydroxyl group is consistent with the simulation data of possible TP-terpolymer structures [39]. Stretching vibrations of the C=C bond of the TP aromatic nucleus are observed in the range of 1610–1580 cm^−1^ (Figure S8b). The absorption bands at 1097 and 813 cm^−1^ relate to the tetrahydropyran cycle, and the absorption band at 1169 cm^−1^ is a concern to the tocol fragment. Figure S8 shows the IR spectra of TP composition powders as well. Here, the terpolymer absorption bands are visible only; the TP’s most intense bands were not found in them, apparently due to their low content. However, signals of drug protons are visible in the ^1^H NMR spectra of the TP-NP_S;_ most of them overlap with those of the terpolymers, and only the CH_3_ groups of the TP molecule stand apart.

In work [39], TP was encapsulated into amphiphilic VP-TEGDM copolymer and VP-HMA-TEGDM terpolymer (FB7) as a member of the VP family of terpolymers. The antiradical activity of these TP-NPs was studied in vitro by luminol-dependent chemiluminescence, and the free radical content in a mouse brain homogenate [58] was evaluated. The effect of FB7, TP-FB7, as well as the initial TP on the TBARS accumulation during spontaneous lipid peroxidation (LPO) in the mouse brain homogenates were examined [39] in accordance with the method [59]. The observed difference of half-maximal inhibition IC_50_ in the TBARS accumulation process for TP-FB7 and TP was due to the slow release of TP from the polymer particles. This is supported by the kinetics of TBARS accumulation in the presence of TP and TP-FB7 [39]. These results were associated with the release of TP from polymer particles when added to the homogenate. In the work [37], we have shown that the fluorescence signal of methyl pheophorbide *a* (MPP) encapsulated in FB12 increased gradually under incubating with liposomes and tissue homogenate. Thus, TP and MPP molecules were released from the NPs based on FB7 and FB12 terpolymers when interacting with biological objects.

In this work, we used other approaches to estimate the antiradical activity of initial and encapsulated TP. The radical scavenging capacity of native TP and water-soluble TP forms was evaluated using 2,2′-azino-bis(3-ethylbenzothiazoline-6-sulphonic acid) (ABTS) and 2,2-diphenyl-1-picrylhydrazyl (DPPH) assays. It is known that compounds with antiradical properties reduce ABTS^•+^ radical cation to the initial structure ABTS (electron transfer) [60,61,62] or transfer a hydrogen atom to form ABTSH (atom transfer hydrogen) [62,63]. As a result of ABTS^•+^ reduction, the absorbance of the characteristic band of the radical cation [64] in the spectrum decreases. Trolox is used as a reference antioxidant. The results were expressed as IC_50_ and TEAC values (Trolox equivalent antioxidant capacity) calculated by dividing the slope of ABTS^•+^ concentration decrease vs. the antioxidant concentration by the slope of the Trolox plot).

This reaction was studied in ethanol in the presence of native TP and encapsulated TP. Our studies of alcoholic solutions of TP-NPs by the DLS method showed that when aqueous solutions of TP-NPs were added to ethanol, the aggregative structure of the solution could be changed. In contrast to water, the size of the scattering centers was ~5–6 nm and ~17–20 nm, and the average light scattering intensity decreased by two orders of magnitude. Thus, aggregation of TP-NPs is suppressed in ethanol, and individual macromolecules and small aggregates are present in the solution. This is supported by the absorption spectra of transparent TP-NP solutions in ethanol (Figure 13b).

The analysis of the absorption spectra of mixtures of ethanol–water solution of TP-NPs (1:1 vol.) showed that the optical density of the absorption band of TP changes in proportion to dilution; it means that its molecules stay encapsulated in NPs in these mixtures. As an example, Figure S9 shows the absorption spectra of an aqueous solution of TP-FB12 in water and its mixture with ethanol.

**Figure 16:**
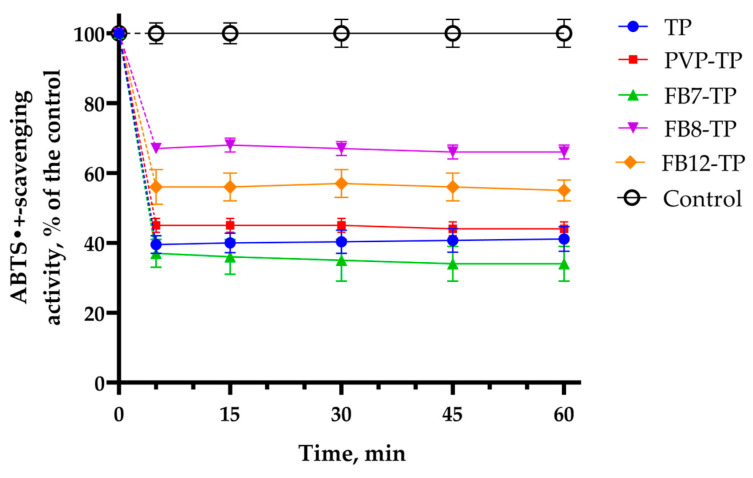
Effect of the studied compounds (at the concentration 20 µM) on the decolorization reaction of ABTS^•+^. The results were presented as a percentage of the control (sample with ABTS^•+^ without tested compounds).

The influence of the initial TP and TP-NPs on the ABTS^•+^ decolorization (i.e., activity) as a function of time was studied (Figure 16). It can be seen that the decolorization reaction of ABTS^•+^ under the action of all the studied compounds proceeds rapidly. The ABTS^•+^ radical cations are reduced to the original ABTS structure after only 5 min. The antiradical activity of each object under study does not change with a further increase in the reaction time. Thus, the reduction of the ABTS^•+^ radical cations proceeds at a high rate. This may be because the ABTS^•+^ radical cation reacts both with TP molecules adsorbed on the surface of NPs and encapsulated in NPs as a result of its diffusion into polymer structures.

It follows from the presented data (Figure 16) that the efficiency of reduction of ABTS^•+^ radical cations depends on the type of TP-NPs. We observe the maximum activity of TP itself and TP-FB7 at a concentration of 20 µM. The activity of PVP-TP is lower than that of TP-FB7 but is also comparable to TP. We observe the lowest antiradical activity of TP encapsulated in FB8 and FB12 nanoparticles.

Table 3 shows the IC_50_ values for the decolorization reaction of ABTS^•+^ in the presence of initial and encapsulated TP. All studied TP-NP structures have antioxidant activity. TP-PVP and TP-FB7 show similar activity to the native TP. PVP has no effect on the ABTS radical in the concentration range of 1–10 µM; at a concentration of 50 μM, it reduces the intensity of the optical density of the radical by ~6% (94 ± 3% of the activity of the ABTS radical is retained), and at a concentration of 100 μM, 92 ± 1% of the activity of the ABTS radical is retained. Unlike PVP, FB7 has no effect on the ABTS radical in the concentration range from 1–100 µM. The lipophilicity of FB7 macromolecules prevents the adsorption of ABTS radicals.

It can be seen from Table 3 that the IC_50_ values of the decolorization reaction of the ABTS^•+^ radical cation in the presence of TP-FB8 and TP-FB12 are higher, and the activity is lower than that of the initial TP and FB7-TP. All TP-NP structures showed TEAC values similar to native TP (Table 3).

The activity of initial and encapsulated TP was also determined by the reaction of the stable radical DPPH, a hydrogen acceptor concerning antioxidants hydrogen donors. The IC_50_ values of the reaction with native TP and its water-soluble forms are also shown in Table 3. It can be seen that FB7 and PVP had no effect on the DPPH radical in the concentration range from 1 to 100 and from 1 to 50 μM, respectively. The IC_50_ values for the original TP and water-soluble copolymers TP-FB7 do not differ significantly. Thus, the DPPH method showed that the antiradical activity of TP-FB7 is retained at the level of the initial tocopherol. The IC_50_ values for TP-FB8, TP-FB12, and TP-PVP are higher than those of the original TP and TP-FB7.

The interaction efficiency of TP with ABTS^•+^ radical cations and DPPH radicals may be due to the size of NPs and diffusion limitations for their penetration into such structures. As shown above, the sizes of NPs based on FB8 and FB12 in ethanol are 3–4 times larger than those of FB7. Significant diffusion restrictions for radical interactions can also arise in individual FB8 and FB12 macromolecules due to their topology and localization of TP molecules into a region with a high packing density. Due to their branched structure, the FB8 and FB12 terpolymers are characterized by a high molecular packing density and a high content of methacrylate units, which form low-polarity regions in which hydrophobic TP molecules can be localized. Thus, using the ABTS and DPPH methods, it is possible to evaluate the antioxidant activity of TP encapsulated in NPs based on terpolymers of different structures and to select water-soluble structures based on a low-toxicity terpolymer that exhibits higher antioxidant.

## 3. Materials and Methods

### 3.1. Synthesis of N-Vinylpyrrolidone with (Di)methacrylates Terpolymers

*N*-vinylpyrrolidone (99%, stabilized, Acros Organics) was purified by vacuum distillation. AlkMA comonomers such as HMA, CHMA, and TEGDM (95%, Aldrich, St. Louis, MI, USA), solvents: toluene (analytical grade), *n*-hexane and chloroform (both chemically pure) were used as received. 2,2′-Azo-bis-isobutyronitrile (AIBN) as the radical copolymerization initiator was purified by recrystallization from ethanol.

Each synthesis was carried out in a three-necked flask equipped with a reflux condenser and a thermometer with continuous bubbling with argon for 2 h at 80 °C in a thermostat. VP-HMA-TEGDM and VP-CHMA-TEGDM terpolymers have been prepared at molar monomer content: 99:1:2 (FB9), 98:2:2 (FB7), 98:2:5 (FB8, FB12). All components of the reaction mixture were introduced simultaneously. The comonomer content in toluene was ~20 wt%. The AIBN concentration in the solution is 0.02 mol L^−1^. After completion of the reaction, toluene-soluble products were obtained; the reaction mixtures were clear and homogeneous. The terpolymers were isolated by precipitation using a tenfold excess of the precipitant, *n*-hexane. The precipitates were filtered on a Buchner funnel and dried from the toluene/*n*-hexane to constant weight in air and in vacuo. The yields of the main fraction of terpolymers are indicated in Table 1.

The terpolymers were further purified by reprecipitation for IR and NMR spectroscopy studies. To do this, the terpolymers were dissolved in chloroform, precipitated with *n*-hexane, and dried in air and in vacuo.

### 3.2. TP Encapsulation into NPs and Its Antiradical Activity

TP was encapsulated into FB7, FB8, and FB12 nanoparticles, according to the method [39]. For comparison, TP-PVP structure was prepared, too. The TP content per terpolymer or PVP was 3.7 wt%. Water-soluble TP-NPs were prepared in two steps. Encapsulation of the drug into terpolymer particles was carried out using solutions of the terpolymers and TP in isopropyl alcohol. For this, TP solution was added dropwise to the terpolymer solutions under constant stirring at room temperature. After drying from isopropyl alcohol in air and in vacuo, the polymer films containing TP were dissolved in water. To prepare the TP-NPs, 100 mL of a terpolymer solution in isopropyl alcohol (7 mg mL^−1^) and 18 mL of TP in isopropyl alcohol (1.4 mg mL^−1^) were used.

The antioxidant activity of TP and TP-NPs structures was screened using the ABTS^•+^-Scavenging Activity method [65]. When ABTS is oxidized with potassium persulfate in water, the monocation radical 2,2′-azino-bis(3-ethylbenzthiazoline-6-sulfonic acid) (ABTS^•+^) is formed, which is reduced in the presence of hydrogen-donating antioxidants. An aqueous solution of ABTS^•+^ is added to ethanol containing TP or its water-soluble form, and a decrease in the absorption intensity of the cation radical at a wavelength of 743 nm during interaction with an antioxidant is recorded.

The ability of initial and encapsulated TP to scavenge radicals was also evaluated using a method based on the interaction of an antioxidant with a stable chromogen radical DPPH (2,2′-diphenyl-1-picrylhydrazyl). The analysis was performed according to the method described by Singh et al. [66]. The sample (1.2 mL) contained an aliquot of 10 µL of the substance under study, ethanol, and 1 mL of a solution containing the DPPH radical in ethanol (85 µM). The samples were incubated for 30 min at 30 °C in the dark, and the optical density of the solution was measured at 517 nm.

Trolox was used as a positive control. The results were presented as a percentage of the control. For the compounds, we also determined the IC_50_ values (compound concentration required for 50% reduction of the ABTS radical). Antioxidant capacity as Trolox equivalent (TEAC) values were determined as the ratio between the slopes obtained from the linear correlation of the ABTS radical absorbance with the concentrations of tested compounds and Trolox.

### 3.3. The Methods

#### 3.3.1. Elemental Analysis

The nitrogen atom content in terpolymers was determined by elemental analysis on a CHNS/O instrument Vario MICRO cube (Elementar Analysensysteme GmbH, Langenselbold, Germany, 2007), and it was 10–11 wt%.

#### 3.3.2. IR- and ^1^H NMR, ^13^C NMR Spectroscopy

IR- and ^1^H NMR-spectroscopy were used to identify the molecular structure of the studied terpolymers and TP-terpolymer compositions in solid state and in solution. The IR spectra were recorded on a Bruker α FTIR instrument in transmission mode. NMR experiments were performed on a Bruker Avance III spectrometer with an 11.7 T superconducting magnet. The resonance frequencies for ^1^H and ^13^C nuclei were 500.2 and 125.8 MHz, respectively. Experiments were performed at room temperature in CDCl_3_ solutions. Residual signals of the solvent were used for chemical shift calibration: 7.24 ppm in ^1^H and 77.23 in ^13^C spectra. The assignment of the signals was performed using ^1^H, ^13^C, and ^1^H-^13^C HSQC spectra and data published in the literature [43,67,68,69]. ^13^C spectra used to estimate the composition of copolymers were obtained using the 30° pulse and ^1^H decoupling during the acquisition (1 s) and relaxation delay (1 s) with 18,432 transients. An exponential line broadening of 10 Hz was applied.

#### 3.3.3. Size-exclusion Chromatography

The absolute molecular weights *M*_w_ and polydispersity *PD* = *M*_w_/*M*_n_ of the studied terpolymers were determined by SEC using a Waters liquid chromatograph (2 columns PS-gel, 5 μm, MIXED-C, 300 × 7.5 mm) (Waters, Milford, MA, USA). It was equipped with a refractive index detector and a multiangle light scattering detector, WYATT DAWN HELEOS II, Wyatt, 658 nm. The eluent was *N*-methylpyrrolidone with lithium chloride addition (0.5 wt%) to avoid terpolymer aggregation. The temperature was 70 °C, and the elution rate was 1 mL min^−1^. The d*n*/d*c* values were determined from multiangle light scattering detector data (MALS). All terpolymer solutions (20 mg mL^−1^) were preliminarily filtered; the pore diameter of the filters was 0.2 μm. Astra software version 5.3.2.20T was used to calculate the absolute weight average molecular weight of the studied terpolymers from MALS data.

#### 3.3.4. Dynamic Light Scattering

The hydrodynamic radii, *R*_h_, of terpolymers in water were determined by dynamic light scattering (DLS). Before preparing samples for the measurements, the solutions were filtered using a filter with a pore diameter of 0.45 μm, and the vials with the solution were thermostated at a given temperature for 20 min. The temperature range for measurements was 25–42 °C. The DLS measurements were carried out using a Photocor Compact instrument (Photocor LTD, RF) equipped with a diode laser operating at a wavelength of 654 nm. Solutions of terpolymers were analyzed at a detection angle of 90°. The experimental data were processed using the DynaLS software, version 2.8.3. The size distribution curves for scattering centers were obtained by processing the results of measurements of scattering intensity fluctuations by solutions. The terpolymers’ hydrodynamic radii *R*_h_ were calculated by the Einstein–Stokes equation.

#### 3.3.5. TEM Study of FB7, FB8, FB12 and TP-FB7

TEM images of the studied terpolymers and TP water-soluble forms were obtained from water solutions using the JEM-2100 microscope operating at 200 kV. For the study, solutions were purified from large particles and impurities by filtration (PES filter, 0.45 µm). A drop of solution was applied to a copper grid covered with a carbon film and dried on air at room temperature. Under these conditions, water strongly bound to the polymers was apparently retained in the samples.

#### 3.3.6. TG and DSC Studies

The thermo-chemical properties of the prepared terpolymers were studied by thermogravimetry (TG) and differential scanning calorimetry (DSC). The thermal effects and glass transition temperatures were determined at the third heating cycle in the temperature range of 30–180 °C to remove volatile products (water and solvent residues) from the samples. TG studies were performed in the range of 30–500 °C. All measurements were made on the NETZSCH STA 449F3 at a heating rate of 5 K min^−1^ in helium.

#### 3.3.7. Electronic Absorption Spectroscopy

The absorption spectra of TP-NPs were recorded using Specord M40 spectrometer (Karl Zeiss, Jena, Germany); the cuvette was 1 cm. The absorption spectra of the cation radical ABTS^•+^ at the wavelength of 743 nm and DPPH radical at the wavelength of 517 nm during their interactions with an antioxidant were recorded using an Agilent Cary 60 UV–vis spectrophotometer (Agilent, Santa Clara, CA, USA).

#### 3.3.8. Study of the Cytotoxicity of Terpolymers on Normal and Tumor Cells

The cytotoxicity study in vitro of TP terpolymer compositions was carried out on normal Vero cells (renal epithelium of the African green monkey) and tumor HeLa cells (human cervical adenocarcinoma, clone M-HeLa) using the MTT test. The cultures were obtained from the collection of the Institute of Cytology of the Russian Academy of Sciences (Institute of Cytology of Russian Academy of Sciences, St. Petersburg, Russia). HeLa cells were grown in Eagle’s MEM medium, Vero cells in DMEM medium (PanEco, Russia) containing 10% fetal calf serum (NeoFroxx, Гepмaния), penicillin (50 U mL^−1^), streptomycin (50 μg mL^−1^) in atmosphere of 5% CO_2_ at 37 °C.

For experiments, the cells were seeded into 96-well culture plates at a concentration of 5 × 10^4^ cells mL^−1^. After 24 h, terpolymers were added into the wells in a concentration range from 0.08 ÷ 5.00 mg mL^−1^. Before the experiment, terpolymers were dissolved in the culture medium. After 72 h exposure, 3-(4,5-dimethylthiazol-2-yl)-2,5-diphenyl-2H-tetrazolium bromide (MTT) was added to the incubation medium at a concentration of 0.5 mg mL^−1^ for 4 h. Then, the medium was aspirated, and the resulting formazan crystals were dissolved in 100% DMSO. The optical density was measured at a fundamental wavelength of 570 nm and a background wavelength of 620 nm using a Spark 10M multifunctional microplate reader (Tecan, Männedorf, Switzerland).

## 4. Conclusions

Thus, in this work, we have shown that amphiphilic terpolymers based on biocompatible *N*-vinylpyrrolidone with different topological structures can be synthesized depending on the composition of the monomer mixture by a simple and efficient one-step method. When the content of TEGDM in the reaction mixture was 2 mol%, nanogels with a cyclic structure and absolute molecular weight of about 35 and 41 kDa were obtained. After increasing the content of TEGDM from 2 to 5 mol% in the reaction mixture, branched terpolymers with a molecular weight of more than 100 kDa were formed. The tendency of amphiphilic terpolymers to form aggregates in water was shown, and the dependence of their hydrodynamic radii on the composition of macromolecules and their topology was established. According to TEM data, the studied terpolymers are amorphous. However, regions of high order were found in some of them; their appearance may be associated with a change in the conformation of PVP chains due to an interaction with water molecules and their convergence. None of the terpolymers demonstrated cytotoxic effects for noncancerous Vero and tumor HeLa cells. Hydrophobic effective antioxidant, D-α-tocopherol, was encapsulated into NPs based on VP-AlkMA-TEGDM terpolymers, and its antiradical activity was studied by ABTS (monocation radical 2,2′-azino-bis(3-ethylbenzthiazoline-6-sulfonic acid) and DPPH (2,2′-diphenyl-1-picrylhydrazyl) methods. An influence of the initial TP and TP-NPs on the ABTS^•+^ decolorization as a function of time was studied. IC_50_ and TEAC values were determined using radical labels. The studied terpolymers based on biocompatible *N*-vinylpyrrolidone can be of interest as new platforms for insoluble and poorly soluble in water biologically active compounds to increase their solubility and bioavailability.

## Figures and Tables

**Table 1:** Compositions of initial reaction mixtures and terpolymers calculated from *N* atom content in them and their yields.

Terpolymers	Monomer Mixtures	Molar Ratio of VP-AlkMA-TEGDM Comonomers	Main Fraction Yield, %	*N* Atom Content in Terpolymers, %	[VP]:[AlkMA-TEGDM] in Terpolymers, mol%
FB9	VP-HMA-TEGDM	99:1:2	96.0	10.9	93.3:6.7
FB7	VP-HMA-TEGDM	98:2:2	95.0	11.0	96.6:3.4
FB8	VP-HMA-TEGDM	98:2:5	95.0	10.4	91.4:8.6
FB12	VP-CHMA-TEGDM	98:2:5	97.0	10.3	90.9:9.1

**Table 2:** Molecular weight characteristics of VP-AlkMA-TEGDM terpolymers and their critical concentrations of aggregation (CAC) in water.

Terpolymers	*M*_w_, kDa	*PD*	d*n*/d*c ^b^*	CAC in Water, mg mL^−1^
FB9	41.0/39.4 *^a^*	1.7/1.8	0.051/0.051	0.57
FB7	34.8/50.1	1.6/2.0	0.052/0.051	0.30
FB8	160.0/132.8	4.8/4.9	0.046/0.053	0.46
FB12	113.0/108.6	3.0/4.5	0.045/0.056	0.55

*^a^* The numerator and the denominator indicate the characteristics of the initial terpolymers and those after purification, respectively. *^b^* Refractive index increment of terpolymers in *N*-methylpyrrolidone.

**Table 3:** Effect of the studied compounds on the decolorization reaction of ABTS^•+^ and with respect to DPPH-radicals.

Compounds	ABTS^•+^-Scavenging Activity,		DPPH Inhibition, IC_50_ (µM)
	IC_50_ (µM)	TEAC ^1^	
TP	16.0 ± 1.1	0.96 ± 0.06	16.08 ± 0.8
TP-PVP	18.5 ± 1.6	1.03 ± 0.04	24.4 ± 2.4
TP-FB7	15.8 ± 0.7	0.99 ± 0.02	14.4 ± 1.9
TP-FB8	26.2 ± 1.5	1.02 ± 0.03	22.7 ± 1.9
TP-FB12	26.8 ± 0.9	1.03 ± 0.02	21.1 ± 1.9
PVP	- *	- *	- *
FB7	- *	- *	- *

*—does not show activity in the range of concentrations. ^1^ TEAC (Trolox equivalent antioxidant capacity) was determined from the ratio of the slopes of the concentration-response curves test compound/Trolox.

## Data Availability

Not applicable.

## Supplementary Materials

The following supporting information can be downloaded at https://www.mdpi.com/article/10.3390/ijms242015170/s1.
