# Supplementary material for: Macromolecular Design and Engineering of New Amphiphilic N-Vinylpyrrolidone Terpolymers for Biomedical Applications
Source: Int J Mol Sci. 2023 Oct 14;24(20):15170. doi: 10.3390/ijms242015170 (PMC10607074; doi:10.3390/ijms242015170)
Supplement: Supplementary file 1 [file ijms-24-15170-s001.zip › ijms-2629049-supplementary.pdf]

International Journal of *Molecular Sciences*

MDPI

Supplementary Material

Macromolecular Design and Engineering of New Amphiphilic *N*-Vinylpyrrolidone Terpolymers for Biomedical Applications

Svetlana V. Kurmaz [1,*], Evgenia O. Perepelitsina [1], Sergey G. Vasiliev [1], Irina A. Avilova [1], Igor I. Khodos [2], Vladimir A. Kurmaz [1], Dmitry A. Chernyaev [1], Yuliya V. Soldatova [1], Natalia V. Filatova [1] and Irina I. Faingold [1]

[1] Federal Research Center of Problems of Chemical Physics and Medicinal Chemistry, Russian Academy of Sciences, 142432 Chernogolovka, Russia; jane@icp.ac.ru (E.O.P.); viesssw@mail.ru (S.G.V.); irkaavka@gmail.com (I.A.A.); kurmaz@icp.ac.ru (V.A.K.); chernyayevda@icp.ac.ru (D.A.C.); soldatovayv@gmail.com (Y.V.S.); natasha55555@yandex.ru (N.V.F.); ifaingold@mail.ru (I.I.F.)

[2] Institute of Microelectronics Technology and High-Purity Materials, Russian Academy of Sciences, 142432 Chernogolovka, Russia; khodos.igor@mail.ru

* Correspondence: skurmaz@icp.ac.ru; Tel.: +7-496-522-10-89

Citation: Kurmaz, S.V.; Perepelitsina, E.O.; Vasiliev, S.G.; Avilova, I.A.; Khodos, I.I.; Kurmaz, V.A.; Chernyaev, D.A.; Soldatova, Y.V.; Filatova, N.V.; Faingold, I.I. Macromolecular Design and Engineering of New Amphiphilic *N*-Vinylpyrrolidone Terpolymers for Biomedical Applications. *Int. J. Mol. Sci.* **2023**, *24*, 15170. https://doi.org/10.3390/ijms242015170

Academic Editor: Ilya Nifant'ev

Received: 8 September 2023
Revised: 6 October 2023
Accepted: 11 October 2023
Published: 14 October 2023

Copyright: © 2023 by the authors. Submitted for possible open access publication under the terms and conditions of the Creative Commons Attribution (CC BY) license (https://creativecommons.org/licenses/by/4.0/).

Figure S1: The region of O-CH_2 and O-CH groups in ^{13}C NMR spectra of terpolymers used for the composition calculation.

Figure S2: Molecular weight distribution curves of the terpolymers and linear PVP.

Figure S3: MALS traces (a) and dependencies of macromolecules root mean square (rms) of radius gyration on (b) M and (c) V_R for the studied terpolymers and linear PVP.

Figure S4: Semilogarithmic dependencies of light scattering intensity *I* on the terpolymer concentration in water at 22 °C.

Figure S5: Mass (MD) and number (ND) distribution on particle size (**a**) in FB7 and (**b**) FB8 water solution at different temperatures. [FB7] = 0.31 mg ml^{-1}, [FB8] = 0.31 mg ml^{-1}.

Figure S6: DSC curves of terpolymers FB8 at 1—3 heating cycle.

Figure S7: TG curves for the studied terpolymers at low degrees of conversion.

Figure S8: FTIR spectra of TP-FB7, TP-FB8 and TP-FB12, TP-PVP, TP in the regions: (**a**) of 4000—2400 cm^{-1} and (b) 1800—400 cm^{-1}.

Figure S9: Absorption spectra of TP-FB12 in water and water/ethanol mixture; cuvette is 1 cm.

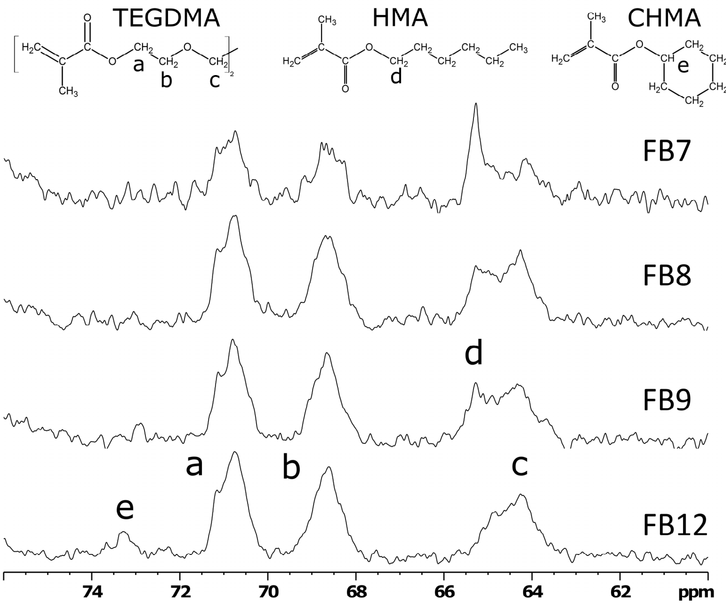

Figure S1: The region of O-CH2 and O-CH groups in ^{13}C NMR spectra of terpolymers used for the calculation of the composition.

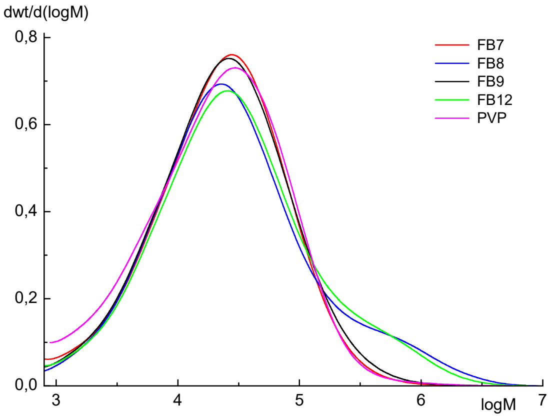

Figure S2: Molecular weight distribution curves of the terpolymers and linear PVP.

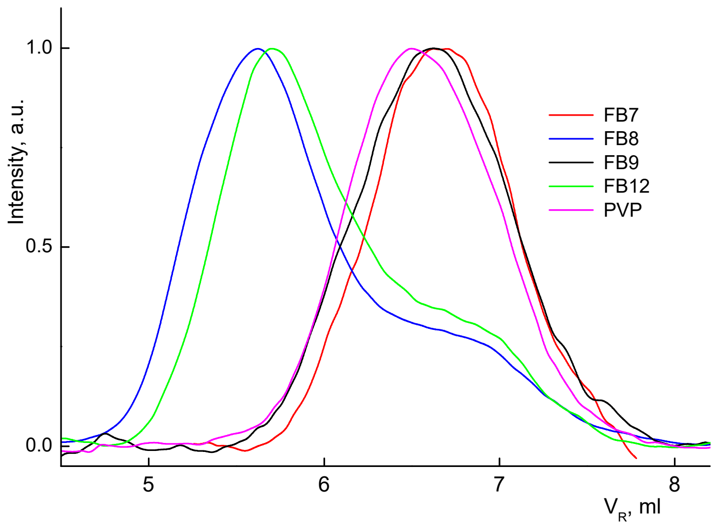
1.0
0.5
0.0
Intensity, a.u.
FB7
FB8
FB9
FB12
PVP
5
6
7
8
V_R, ml

(**a**)

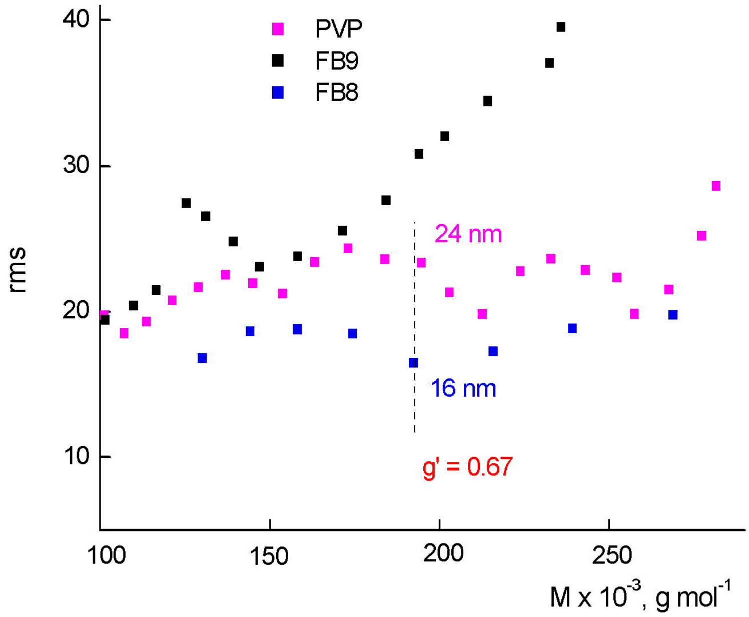
40
30
20
10
rms
PVP
FB9
FB8
24 nm
16 nm
g' = 0.67
100
150
200
250
M x 10-3, g mol-1

(**b**)

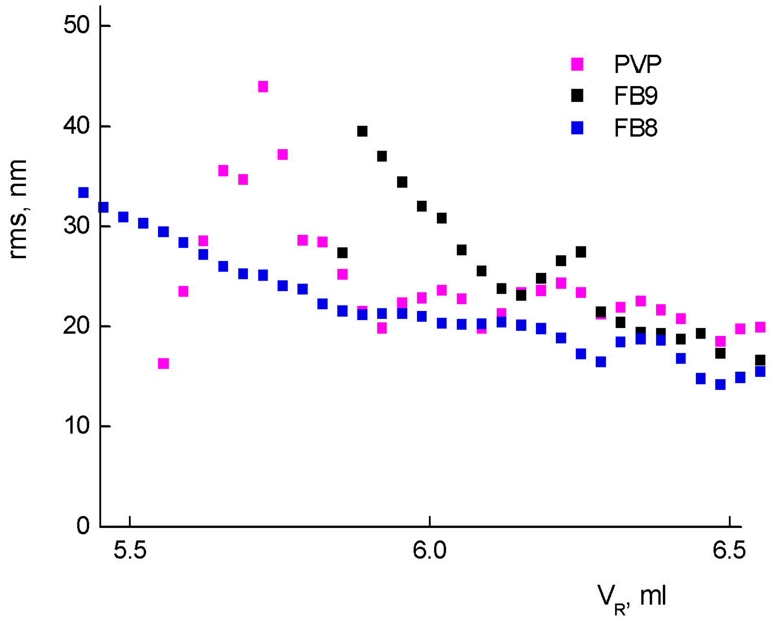

(**c**)

Figure S3: MALS traces (**a**) and dependencies of macromolecules root mean square (rms) of radius gyration on (**b**) *M* and (**c**) V_R for the studied terpolymers and linear PVP.

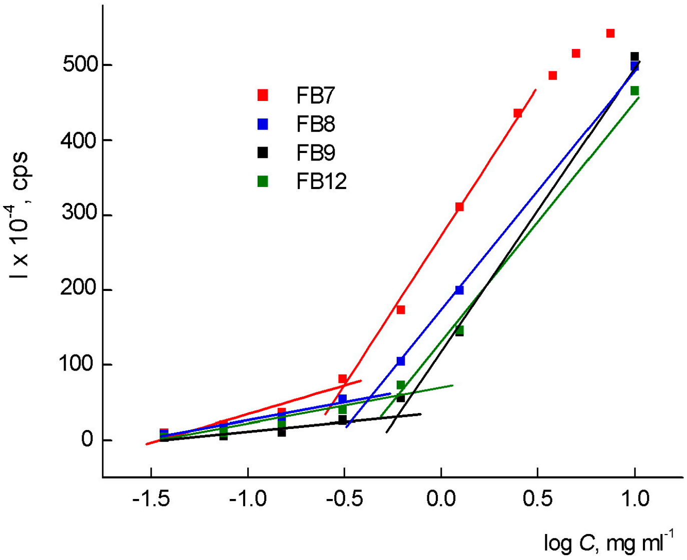

Figure S4: Semilogarithmic dependencies of light scattering intensity *I* on the terpolymer concentration in water at 22 °C.

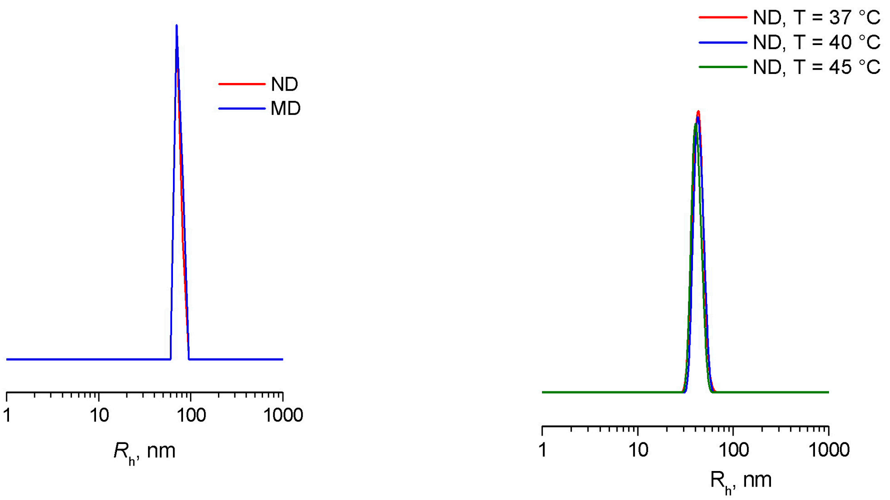

(**a**) (**b**)

Figure S5: Mass (MD) and number (ND) distribution on particle size (**a**) in FB7 and (**b**) FB8 water solution at different temperatures. [FB7] = 0.31 mg ml^{-1}, [FB8] = 0.31 mg ml^{-1}.

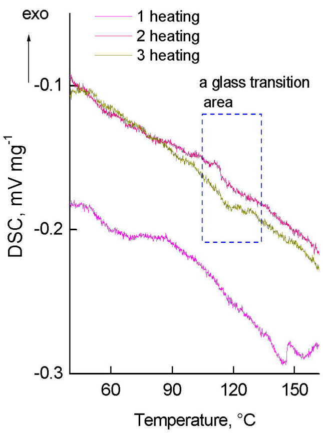

(**a**)

Figure S6: DSC curves of terpolymers FB8 at 1—3 heating cycle.

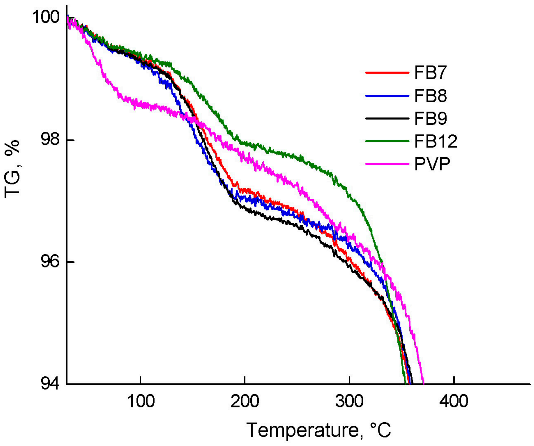

Figure S7: TG curves for the studied terpolymers at low degrees of conversion.

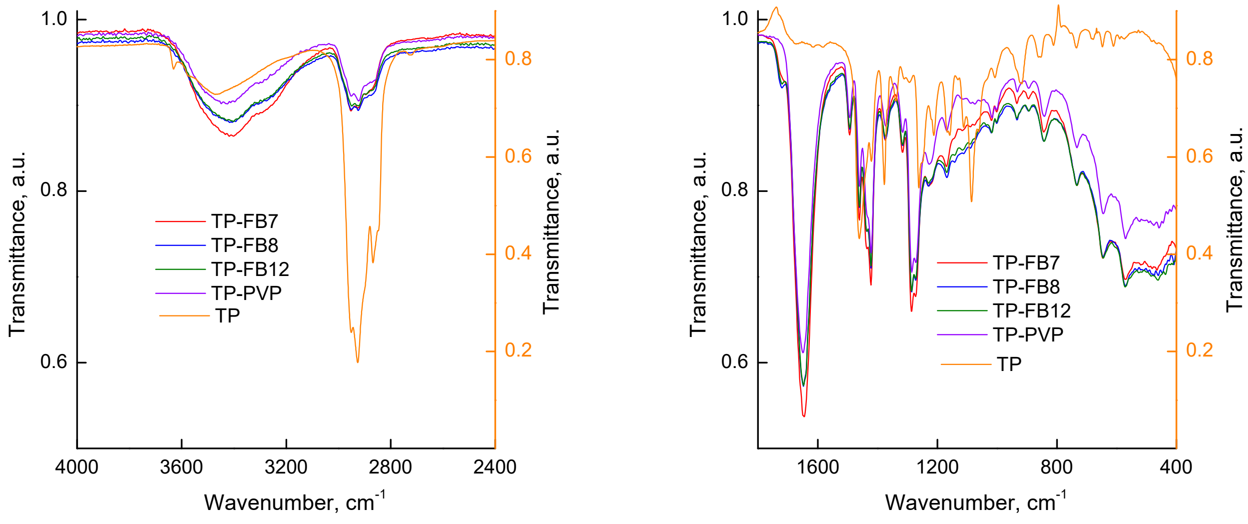

(**a**) (**b**)

Figure S8: FTIR spectra of TP-FB7, TP-FB8 and TP-FB12, TP-PVP powders, and TP in the regions: (a) of 4000—2400 cm^{-1} and (b) 1800—400 cm^{-1}.

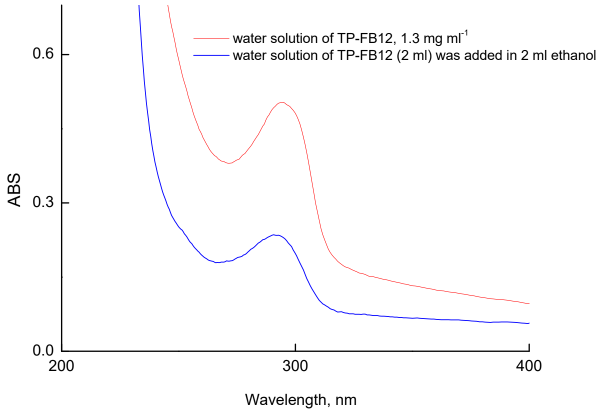

Figure S9: Absorption spectra of TP-FB12 in water and water/ethanol mixture; cuvette is 1 cm.
